# Development of a Rapid LC-MS/MS Method for the Determination of Emerging *Fusarium* mycotoxins Enniatins and Beauvericin in Human Biological Fluids

**DOI:** 10.3390/toxins7093554

**Published:** 2015-09-09

**Authors:** Ana Belén Serrano, Anna Laura Capriotti, Chiara Cavaliere, Susy Piovesana, Roberto Samperi, Salvatore Ventura, Aldo Laganà

**Affiliations:** 1 Laboratorio de Toxicología, Departament de Medicina Preventiva I Salut Pública, Ciències de l’Alimentació, Toxicologia I Medicina Legal Facultat de Farmàcia, Universitat de València, València 46010, Spain; E-Mail: A.Belen.Serrano@uv.es; 2 Department of Chemistry, Università di Roma “La Sapienza”, Piazzale Aldo Moro 5, Rome 00185, Italy; E-Mails: annalaura.capriotti@uniroma1.it (A.L.C.); susy.piovesana@uniroma1.it (S.P.); roberto.samperi@uniroma1.it (R.S.); salvatore.ventura@uniroma1.it (S.V.); aldo.lagana@uniroma1.it (A.L.)

**Keywords:** mycotoxins, enniatins, beauvericin, urine, plasma, liquid chromatography-tandem mass spectrometry

## Abstract

A novel method for the simultaneous determination of enniatins A, A1, B and B1 and beauvericin, both in human urine and plasma samples, was developed and validated. The method consisted of a simple and easy pretreatment, specific for each matrix, followed by solid phase extraction (SPE) and detection by high performance liquid chromatography-tandem mass spectrometry with an electrospray ion source. The optimized SPE method was performed on graphitized carbon black cartridges after suitable dilution of the extracts, which allowed high mycotoxin absolute recoveries (76%–103%) and the removal of the major interferences from the matrix. The method was extensively evaluated for plasma and urine samples separately, providing satisfactory results in terms of linearity (*R*^2^ of 0.991–0.999), process efficiency (>81%), trueness (recoveries between 85% and 120%), intra-day precision (relative standard deviation, RSD < 18%), inter-day precision (RSD < 21%) and method quantification limits (ranging between 20 ng·L^−1^ and 40 ng·L^−1^ in plasma and between 5 ng·L^−1^ and 20 ng·L^−1^ in urine). Finally, the highly sensitive validated method was applied to some urine and plasma samples from different donors.

## 1. Introduction

Mycotoxins present a wide range of adverse effects for consumer health, including carcinogenic, mutagenic, estrogenic and immunosuppressive effects [1]. Among the genera capable of producing mycotoxins in several commodities [2], *Fusarium* species are probably the most prevalent toxin-producing fungi of the temperate regions of America, Europe and Asia [3].

Governmental authorities from different nations have general concerns regarding the harmful effects of mycotoxins on human and animal health. Therefore, maximum levels (MLs) have been set in different food products for mycotoxins with recognized adverse effects, such as trichothecenes A and B, aflatoxins, zearalenone, ochratoxin A, patulin and fumonisins [4]. Furthermore, tolerable daily intake (TDI) or provisional TDI values have been established by the Scientific Committee on Food and the Joint FAO/WHO Expert Committee on Food Additives [5]. In addition to the regulated mycotoxins, currently, attention to the risks posed to human and animal health has also been extended to other potential mycotoxin contaminants, such as the so-called “emerging” *Fusarium mycotoxins*, especially the structurally-related enniatins (ENs) and beauvericin (BEA) [2,6]. The importance of setting legislative measures for ENs and BEA is mainly due to their recently recognized toxicity, as proven by *in vitro* studies on several cell lines [7,8,9], including human cells. For the moment, no reports are available for the toxicity in humans, while *in vivo* preliminary studies on animals showed no observable adverse effects in the treated animals [10,11]. On the other hand, recent studies concerning ENs and BEA occurrence in a wide range of cereal grains (wheat, barley, rye and oat) and their products have been carried out [12,13,14,15]. Results from these studies showed a common mycotoxin co-occurrence, ranging from a few to several thousand mg·kg^−1^. The actions of these co-occurring mycotoxins within the body represent an interesting subject, because synergistic, antagonistic or additive effects could occur. Regarding mycotoxin interactions, Prosperini *et al.* [9] studied the viability of Caco-2 cells evidencing that interactions among different mixtures of ENA, ENA1, ENB and ENB1 could produce a general additive effect.

Regulatory limits have not been established for these fusariotoxins, yet. In this regard, exposure assessment represents the main difficulty, as this cannot be done as long as available data are too limited and methods not accurate enough. Recently, the European Food Safety Authority (EFSA) carried out an assessment of the human risk related to the presence of BEA and ENs in food and feed [16]. For this evaluation, the combined exposure to enniatin A (ENA), enniatin A1 (ENA1), enniatin B (ENB) and enniatin B1 (ENB1) was taken into account. Unfortunately, after an exhaustive review of all of the available information, EFSA could not perform a reliable risk assessment. This is due to the limited data available regarding human and animal exposure, primarily regarding *in vivo* toxicity. Hence, it is important to establish tools for the accurate assessment of human and animal exposure to this group of mycotoxins by determining their levels in body fluids. The only available data indicate that BEA and ENs are absorbed and rapidly metabolized to a range of uncharacterized metabolites [16,17]. Currently, only *in vitro* phase I metabolites of ENB have been established [18], while no information is available either for the other ENs and BEA or for phases II and III. For this reason, until the metabolites are characterized and standards become available, the determination of the parent compounds represents the only accomplishable means.

For the determination and quantification of mycotoxins in complex matrices, analytical methods based on liquid chromatography-tandem mass spectrometry (LC-MS/MS) have been extensively used [2,19,20]. A wide variety of sample preparations, such as liquid-liquid extraction, solid phase extraction (SPE), accelerated solvent extraction, matrix solid-phase dispersion and dilute-and-shoot approaches, have been reported [21].

To the best of the authors’ knowledge, until now, few analytical methodologies have been devoted to the determination and quantification of emerging fusariotoxins and their metabolites in biological fluids [2]. Devreese *et al.* [17] validated the first method for the determination of ENA, ENA1, ENB, ENB1 and BEA in pig plasma by LC-MS/MS with satisfactory results. Recently, Juan *et al.* [22] proposed a new method based on a conventional liquid extraction and determination by LC-MS/MS for the quantitative analysis of ENA in serum, urine and feces from Wistar rats. So far, no method on the determination of ENs, BEA and their metabolites in human biological fluids has been reported in the literature. Given the lipophilic nature of ENs and BEA, it could be easier to find the non-metabolized form of these mycotoxins in plasma than in urine samples. However, this occurrence cannot be excluded *a priori.* The determination of the targeted mycotoxins in urine could be a promising non-invasive alternative.

**Figure 1:**
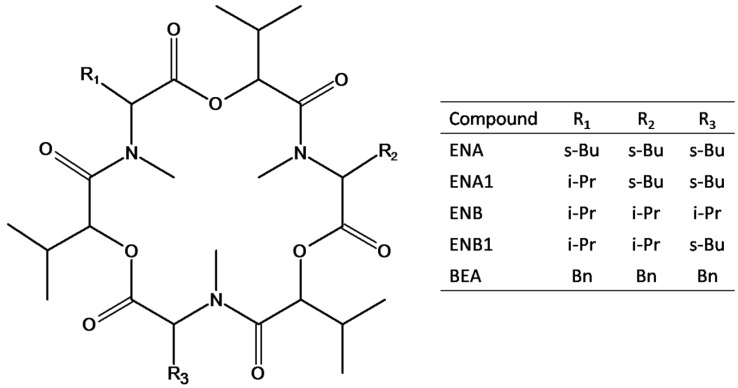
Structures of the investigated mycotoxins, namely enniatin A (ENA), enniatin A1 (ENA1), enniatin B (ENB), enniatin B1 (ENB1) and beauvericin (BEA); R_1_, R_2_, and R_3_ can be sec-butyl (*s*-Bu), isopropyl (*i*-Pr) or benzyl (Bn) groups.

Taking into account the lack of methodology for the extraction of ENs and BEA from human biological fluids, the main aim of this work was the development of a reliable and sensitive analytical method for the simultaneous determination of the main four ENs and BEA (see Figure 1) by LC-MS/MS applicable to human urine and plasma. To achieve this goal, an extraction method specific for the biological fluid, followed by a cleanup based on SPE, was optimized for urine and plasma samples. Finally, after validation, the applicability of the optimized method was demonstrated by the analysis of human samples of urine and plasma.

## 2. Results and Discussion

### 2.1. LC-MS/MS Optimization

A preliminary study was performed to obtain the best instrumental conditions affording high resolution and short analysis time with a suitable analyte separation. Positive and negative ionization modes were tested for all compounds, but all of the mycotoxins gave a better response in positive ionization mode. Sodiated adducts [M+Na]^+^ exhibited higher signal intensities than protonated adducts [M+H]^+^ for all mycotoxins. Sodiated adducts were formed because ENs and BEA are ionophoric compounds capable of forming complexes with monovalent and divalent cations through interactions with carbonyl groups oriented within the molecule. A low amount of Na^+^ may result from the analytical procedure, mainly from the solvents. In general, during the process of fragmentation, the sodiated adducts provide low yields in charged fragments; therefore, they are usually not employed for quantitative purposes. Several authors have reported that sodiated adduct ions can be greatly reduced by adding to the mobile phase modifiers suitable to promote NH_4_^+^ adduct formation [2,23,24,25]. In this sense, in the present work, the addition of ammonium formate and HCOOH to both mobile phases was evaluated. The results indicated that the addition of the above-mentioned modifiers resulted in an enhanced abundance of [M+NH_4_]^+^ and [M+H]^+^ ions. All mycotoxins gave the highest signal intensity employing an LC mobile phase with H_2_O (A) and methanol (MeOH) (B), both with 5 mmol·L^−1^ ammonium formate and 0.1% (*v*/*v*) HCOOH. In these conditions, the [M+NH_4_]^+^ adduct prevailed on [M+H]^+^ formation, and thus, the MS/MS parameters were optimized for each compound in order to select the two most intense transitions of the [M+NH_4_]^+^ adducts. Table 1 shows the list of precursor and product ions of all analytes, as well as their retention time and the optimized S-lens and collision energy (CE). Mycotoxin quantification was performed summing the transitions.

**Table 1:** Retention time, precursor ion, product ions and optimized mass spectrometric parameters for targeted mycotoxins.

Mycotoxin (Abbreviation)	Retention Time (min)	Precursor Ion [M+NH_4_]^+^ (*m*/*z*)	Product Ion (*m*/*z*)	Collision Energy (V)	S-Lens (V)
Enniatin A (ENA)	8.48	699.4	209.7	35	148
			228.0	36	
Enniatin A1 (ENA1)	8.34	685.2	210.0	33	139
			228.0	33	
Enniatin B (ENB)	7.97	657.4	196.0	32	137
			214.0	33	
Enniatin B1 (ENB1)	8.16	671.3	196.0	33	148
			214.0	34	
Beauvericin (BEA)	8.17	801.3	244.0	36	172
			262.0	34	

Apart from the mobile phases used for the separation, other chromatographic parameters were optimized, such as injection volume and column temperature. Injection volume was set to 5 µL, because larger injection volumes increased the matrix effect (ME). Furthermore, analyte separation was improved by testing different column temperatures (25 °C, 30 °C and 40 °C). A thermostated column at 30 °C resulted in a better mycotoxin separation, but ENB1 and BEA were not resolved. This fact does not represent an actual problem, as the two compounds have different molecular weights, and the expected concentrations should not cause ME between them.

### 2.2. Optimization of the Extraction Method

Although LC-MS/MS is a powerful technique and direct analysis or methods with a little sample pretreatment are possible in some cases, results could be affected by a heavy ME, which could lead to low sensitivity. In addition, the presence in biological samples of isobaric interferents giving the same transitions may sometime cause the inaccuracy of the final results. Moreover, dirty extracts can result in progressive column deterioration, as well as signal weakening. Starting from these points, the effect of different factors on the extraction method was examined to develop suitable sample preparation procedures for urine and plasma, according to the individual features of each biological fluid. All parameters were tested by recovery experiments in six replicates at the 50-ng·L^−1^ level for each mycotoxin. Recovery was considered acceptable in the range of 70%–120%.

#### 2.2.1. Urine

A modification of the SPE procedure earlier described by Capriotti *et al.* [14] for the cleanup of a biscuit extract was initially evaluated for the extraction of ENs and BEA from urine. The rationale for the sample dilution and pH adjustment is explained in that reference and the references therein. Briefly, one of the main advantages of using graphitized carbon black (GCB) sorbents is the ability to retain organic analytes from large volumes of water or aqueous samples without breakthrough. However, owing to the relatively low loading capacity of GCB, the presence of other organic substances in the sample at relatively high concentrations can cause the displacement of some low-abundance compounds. Sample dilution and relatively low acidic pH values provide an attenuation of this phenomenon, increasing the recovery of some analytes in many cases. In the present work, the recovery of the selected analytes still increased about 10%–15% by increasing the urine sample dilution with water from 250–500 mL. However, in this way, some interfering compounds that increased blank background and ME were retained and recovered, as well. Suitable modifications that could be tested to tackle this problem and to reduce the presence of interferences are the introduction of a washing step and the tuning of elution volumes. Therefore, we started with the introduction and optimization of a washing step. On the bases of previous experiences [26], MeOH was selected as the best washing solvent to remove interferences without eluting analytes of interest and was tested at three different volumes (2 mL, 4 mL and 5 mL), while the solvent volume for analyte elution was fixed at 10 mL. A washing volume of 2 mL significantly decreased the ME, while larger washing volumes resulted in a significant loss of all of the analytes (recoveries from 63%–82% for 4 mL washing). After optimization of the washing step, we moved on to the elution one. In fact, another critical point of SPE was the elution step, during which the retained analytes were eluted from the sorbent. ENs and BEA were eluted from GCB cartridges using CH_2_Cl_2_/MeOH (80/20, *v*/*v*) containing 0.2% HCOOH [14]. The solvent volume for elution was optimized by testing 5 mL, 10 mL and 15 mL. Recoveries of the analytes increased with increasing eluent volume. However, it was found that with a 15-mL elution volume, recoveries were in the range of 90%–99%, but the ME significantly increased. Therefore, a 10-mL eluent volume was chosen as the best compromise. The entire sample preparation and extraction procedure took about 2 h, including cartridge activation and solvent evaporation, but up to six samples could simultaneously be processed.

After method optimization, the recoveries were compared to those obtained by us employing a recently-published method for the extraction of other mycotoxins in pig urine, based on the technique of salting-out-assisted liquid/liquid extraction (SALLE) [27]. In both cases, the concentration in the samples was 50 ng·L^−1^, and the results are shown in Table 2. As can be seen, for all of the compounds, recoveries obtained by this method were significantly higher than the ones obtained by SALLE.

**Table 2:** Comparison of the recovery (yield) of the proposed method to two published methods for urine and plasma pretreatment. Samples were spiked at 50 ng·L^−1^.

Mycotoxin	Recovery ± RSD ^a^ (%)			
	Urine		Plasma	
	This method	SALLE ^b^ [27]	This method	Deproteinization with ACN [17]
ENA	92 ± 6	85 ± 7	99 ± 7	77 ± 15
ENA1	80 ± 10	64 ± 4	90 ± 3	73 ± 14
ENB	82 ± 1	60 ± 9	97 ± 8	92 ± 7
ENB1	95 ± 4	75 ± 5	76 ± 3	88 ± 6
BEA	87 ± 4	73 ± 11	103 ± 12	62 ± 13

^a^ Relative standard deviation, calculated on six replicates; ^b^ Salting-out-assisted liquid/liquid extraction; the extraction was not tested for the reported mycotoxins in the original work.

#### 2.2.2. Plasma

In early experiments, we tried to prepare the plasma sample by the simplest available approach, according to Devreese *et al.* [17]. Plasma samples, spiked at 50 ng·L^−1^, were deproteinized with acetonitrile (ACN), employed in a 3:1 (*v*/*v*) ratio to plasma. Then, after centrifugation, the supernatant was withdrawn, diluted with 25 mL of water and cleaned up with the GCB cartridge, as already described for urine. However, recoveries obtained by us were not satisfactory for all of the analytes. In order to evaluate the effect of the precipitation step, we carried out a test by adding the analytes to the sample after protein precipitation, obtaining quantitative recoveries (data not shown). A possible explanation for this observation could be that ENs and BEA may be adsorbed by denatured proteins. Following this reasoning, another deproteinizing mixture, consisting of 25 mL of MeOH/H_2_O (40/60, *v*/*v*), was tested. The supernatant recovered after centrifugation was then cleaned up without further manipulations. As shown in Table 2, the 250-µL plasma treatment with 25 mL of MeOH/H_2_O followed by Carbograph cleanup gave better recoveries (76%–103%) than those obtained using the simple ACN/plasma 3:1 (*v*/*v*) protein precipitation (62%–92%).

The entire sample preparation and extraction procedure took slightly more than two hours and allowed simultaneously processing up to six samples.

### 2.3. Method Performance

#### 2.3.1. Linearity, and Process Efficiency

Linearity was tested by evaluation of determination coefficients (*R*^2^). The linear range was estimated for both standard and matrix-matched calibration curves over the range reported in the Experimental Section. Results are summarized in Table 3. Mycotoxin calibration regression lines prepared in solvent, blank urine and blank plasma samples showed excellent *R*^2^ in the ranges 0.991–0.999, 0.991–0.999 and 0.993–0.999, respectively.

**Table 3:** Linearity reported as the determination coefficient (*R*^2^) and process efficiency (%) for targeted mycotoxins in urine and plasma samples. The linear range was estimated for standard and matrix-matched calibration curves over the ranges 0.04–4.0 ng·mL^−1^ (0.2–20 pg injected) and 40–4000 ng·L^−1^, respectively.

Mycotoxin	*R*^2^			Slope of Regression Line (RSD ^a^, %)			Process Efficiency (%) ^b^	
	Solvent	Urine	Plasma	Solvent	Urine	Plasma	Urine	Plasma
ENA	0.998	0.999	0.993	112.4 (1.7)	112.5 (2.3)	116.3 (5.7)	100.0	103.5
ENA1	0.995	0.991	0.994	25.6 (1.4)	26.7 (3.2)	27.9 (5.6)	108.2	109.0
ENB	0.998	0.993	0.994	77.8 (2.0)	77.7 (2.1)	77.6 (4.8)	99.9	99.7
ENB1	0.991	0.995	0.997	65.7 (1.7)	53.7 (2.1)	67.0 (3.8)	81.7	102.0
BEA	0.999	0.998	0.999	118.2 (1.0)	100.0 (2.4)	129.4 (2.4)	84.6	109.5

^a^ Relative standard deviation obtained from the mean of six replicates; ^b^ Process efficiency (%): (slope_matrix−matchedregressionline_/slope_methanolregressionline_) × 100; this is the product of signal enhancement/suppression (matrix effect) and recovery (yield).

As determined by us, the ratio of the slope between matrix-matched and standard calibration lines includes the effects of recovery and the ME, while the relative standard deviation (RSD) represents the effect of individual to individual sample variability.

Very often, when a suitable internal standard is not available, as in this case, the presence of ME is a major drawback for the method performance, because different constituents of biological fluids can lead to a significant suppression or enhancement on the analyte response. Moreover, within the same sample typology, ME variations can be observed from sample to sample. Considering the data shown in Table 2 and Table 3, the products of the two effects, namely process efficiencies (PEs) [28], were within the acceptable range (±20%) both for urine and plasma samples, although the ME in one case was +28%.

#### 2.3.2. Detection and Quantification Limits

When operating in selected reaction monitoring (SRM) mode with the last generation triple quadrupole mass spectrometers, it is quite common to obtain SRM signals without noise. Being so, the calculation of limits of detection (LODs) and limits of quantifications (LOQs) becomes quite challenging. They might be estimated from regression statistics using the standard errors on the intercept coefficient via the formulas: LOD = 3 σ/*S*, and LOQ =10 σ/*S*, as described in the Experimental Section. As this method presupposes the homoscedasticity of variance, the extrapolated concentrations have to be used to prepare spiked samples, which are then analyzed to verify the conformity to the extrapolated value.

Moreover, following the indication and the intendment of the 2002/657/EC [29], three conditions have to be satisfied: First, two SRM transitions have to be considered for identification; second, the relative intensity of the detected ion shall correspond to those of the calibration standard, under certain conditions set within set tolerances; third, it makes no scientific sense to quantify a compound that has not been confirmed. The second condition is rarely taken into account; though, in our experience, sometimes, it becomes the limiting factor. Another question arises from the fact that many authors differentiate between the quantifier (most intense) and qualifier (less intense) transitions. Once more, in our experience, especially at concentrations near the quantification limits, the RSD of the transition sum is lower than the RSD of the most intense one (unless the second transition is much less intense than the first one). This fact has a logical explanation and originates from the smoothing process necessary to obtain a measurable peak for very low concentrations. In addition, as the limiting factor could be the transitions’ intensity ratio, both transitions have to be studied also for detection limits.

**Figure 2:**
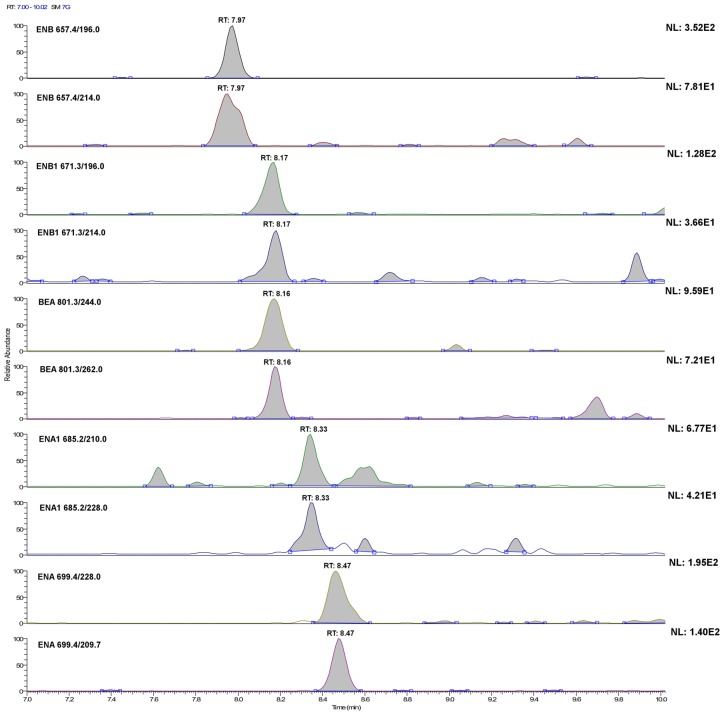
LC-selected reaction monitoring (SRM) chromatograms of the single transitions of a plasma sample extract fortified with the investigated analytes at 20 ng·L^−1^ (acquisition conditions are reported in the Experimental Section). For ENA and BEA, this concentration level is below their method detection limit (MQL), whereas for ENA1, ENB and ENB1, it corresponds to their experimental MQL.

**Figure 3:**
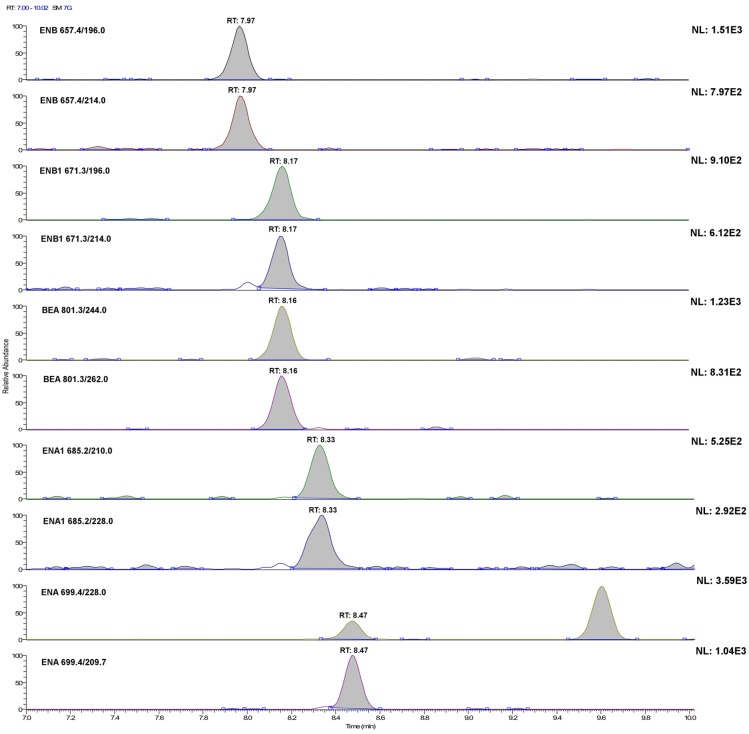
LC-SRM chromatograms of the single transitions of a urine sample extract fortified with the investigated analytes at 20 ng·L^−1^ (acquisition conditions are reported in the Experimental Section). For ENB1, this concentration level corresponds to its method detection limit.

Therefore, for each analyte, the LODs and LOQs were extrapolated as reported in Section 3.5.3, considering the second most intense transition area and the sum of the transition areas, respectively. Then, standard solutions and samples fortified at the extrapolated level were prepared, processed and injected six times. Finally, the dataset was evaluated in terms of RSD of the areas and mean transitions ratio. An acceptable value of 20% of the RSD for quantification limits and 50% for the detection limits was arbitrarily set, whereas the acceptable differences established by 2002/657/EC [29] were considered for the ratio between the areas. When both conditions were respected, a more diluted sample was prepared, whereas a more concentrated sample was tested if not. To avoid repeating the operation too many times, concentration variations were 50%–100%. Results are shown in Table 4. Figure 2 and Figure 3 show, for all of the investigated analytes, the LC-SRM profile of each transition in plasma and urine samples, respectively, fortified at 20 ng·L^−1^. As can be seen, the experimental limits were of the same order of magnitude of the extrapolated ones. In addition, by adding to the matrix-matched calibration line for urine the concentration corresponding to experimental verified method detection limit (MDL), the *R*^2^ did not change significantly. These facts might be due to the restricted concentration range within which calibration lines were considered for expected concentration quantification (the range of linearity covers about three orders of magnitude). In some cases, MDLs and method quantification limits (MQLs) were very similar to each other or even the same: in these cases, for a concentration lower than that evaluated for MDL, they were not in compliance with the provision established by 2002/657/EC [29] regarding the mean value variation of the area ratio between the two transitions. Obviously, the areas of signals present in blanks were subtracted. This fact is important, because the distinction between quantifier and qualifier transition is no longer applicable. Looking at Figure 2 and Figure 3, it should appear clear why, although urine samples were concentrated ten times, whereas plasma samples were not concentrated, MQLs and MDLs are not very different. As can be seen, background signals are more intense for urine. Note that, for some analytes, 20 ng·L^−1^ is near or even under the MDL. This evaluation of MDLs and MQLs should be considered approximate as, although some biological variation was considered, the population was fairly homogeneous in terms of diet. Nevertheless, the limits reported here are five-times lower than those reported for plasma [17] and 500-times lower than those reported for ENA in urine [21].

**Table 4:** Instrumental limit of quantification (ILOQ) and instrumental limit of detection (ILOD), method detection limit (MDL) and method quantification limit (MQL) for urine and plasma, extrapolated (Ext) and experimental (Exp) values.

Mycotoxin	Instrumental				Urine				Plasma			
	ILOQ (pg)		ILOD (pg)		MQL (ng·L^−1^)		MDL (ng·L^−1^)		MQL (ng·L^−1^)		MDL (ng·L^−1^)	
	Ext	Exp	Ext	Exp	Ext	Exp	Ext	Exp	Ext	Exp	Ext	Exp
ENA	0.1	0.2	0.03	0.2	25	10	8	10	65	40	20	40
ENA1	0.5	0.1	0.20	0.05	35	10	10	5	65	20	20	10
ENB	0.1	0.1	0.04	0.05	15	5	5	2.5	55	20	15	10
ENB1	0.1	0.05	0.03	0.05	15	20	5	20	45	20	15	20
BEA	0.3	0.2	0.10	0.05	30	10	8	5	30	40	10	20

#### 2.3.3. Trueness and Precision

According to the European Union Decision 2002/657/EC [29], trueness means “the closeness of agreement between the average value obtained from a large series of test results and an accepted reference value”. However, in the same Decision, it is reported that “When no such certified reference materials (CRMs) are available, it is acceptable that trueness of measurements is assessed through recovery of additions of known amounts of the analyte(s) to a blank matrix”. Because CRMs for ENs and BEA in urine and plasma samples do not exist, the trueness of the method was evaluated by measuring the peak area of the spiked samples and comparing this result to the matrix-matched calibration curve, reported as a percentage, at three concentration levels, *i.e.*, MQL, 2.5 MQL and 10 MQL, whereas the RSD of average recovery was employed to evaluate method precision (intra- and inter-day; *n* = 6). Blanks were subtracted only for samples spiked at the MQL level. Recovery and RSD values regarding urine and plasma samples are shown in Table 5. Following the criteria relying on the 2002/657/EC [29], when trueness and precision are assessed by analyte addition to the matrix, an average recovery ≥90% and a repeatability (RSD) <20% should be obtained. As can be seen, although few recoveries were <90%, none of them were significantly different from the required values.

**Table 5:** Trueness and precision in urine and plasma samples. Trueness was assessed by measuring the peak area of the spiked samples and comparing this result with the matrix-matched calibration curve; the result was expresses as the percentage.

Mycotoxin	Urine						Plasma					
	Trueness % (RSD ^a^)						Trueness % (RSD ^a^)					
	1× MQL		2.5× MQL		10× MQL		1× MQL		2.5× MQL		10× MQL	
	Intra-day	Inter-day	Intra-day	Inter-day	Intra-day	Inter-day	Intra-day	Inter-day	Intra-day	Inter-day	Intra-day	Inter-day
ENA	92 ± 12	88 ± 15	109 ± 8	90 ± 9	102 ± 1	96 ± 14	99 ± 17	101 ± 15	120 ± 6	112 ± 11	95 ± 4	99 ± 11
ENA1	85 ± 10	98 ± 8	96 ± 7	96 ± 11	91 ± 6	94 ± 8	90 ± 13	92 ± 16	114 ± 10	118 ± 14	94 ± 6	93 ± 10
ENB	87 ± 7	110 ± 17	89 ± 13	90 ± 9	98 ± 1	102 ± 10	97 ± 18	95 ± 21	109 ± 12	117 ± 14	110 ± 8	105 ± 9
ENB1	101 ± 14	102 ± 11	89 ± 6	95 ± 8	103 ± 4	90 ± 12	106 ± 13	87 ± 14	112 ± 9	115 ± 9	95 ± 9	98 ± 9
BEA	93 ± 12	97 ± 14	105 ± 10	98 ± 10	101 ± 3	87 ± 12	103 ± 12	91 ± 12	106 ± 10	114 ± 12	96 ± 10	92 ± 11

^a^ RSD, relative standard deviation, obtained from the mean of six replicates.

### 2.4. Application to Samples

The suitability of the method was finally tested by analyzing ten samples of human urine and ten samples of human plasma according to the optimized methods. ENs and BEA were not detected in nine analyzed samples of both plasma and urine, while a trace amount, between MDL and MQL, for ENB1 was detected in a sample of both biological fluids of the same subject.

Until now, mycotoxin occurrence in biological fluids of different animals has been evaluated only in two studies, which were conducted on animals previously treated with a known, relatively large amount of target mycotoxins [17,22]. Therefore, it is obvious that results from the present study related to human fluids were not comparable to those obtained in the above-mentioned studies. On the other hand, the *in vitro* metabolism of emerging *Fusarium* mycotoxins has been rarely studied. At the moment, only phase I metabolism of ENB has been characterized [18]: Ivanova *et al.* identified a total of 12 biotransformation compounds, the oxidation and *N*-demethylation of ENB being the major metabolic pathways. According to [17], there is a vast difference in oral absorption, as well as in the metabolization routes between the various ENs and BEA, although they are structurally similar compounds. Interestingly, ENB1 resulted in being the most absorbed after oral administration to a pig [17].

## 3. Experimental Section

### 3.1. Chemicals and Reagents

ACN, MeOH, CH_2_Cl_2_, ammonium formate (99%), HCOOH (>98%), HCl and MgSO_4_ were supplied by Sigma-Aldrich (Milan, Italy). All reagents were analytical reagent grade; solvents were LC-MS grade. Ultrapure water (resistivity 18.2 MΩ·cm^−1^) was obtained using an Arium water purification system (Sartorious, Florence, Italy). In-house Carbograph cartridges were prepared with 500 mg of Carbograph-4 (surface area of 130 m^2^·g^−1^ and particle size of 120–400 mesh) supplied by LARA (Rome, Italy), while polypropylene tubes and polyethylene frits were supplied by Supelco (Bellefonte, PA, USA). Carbograph cartridges are similar to Carboprep 200 (Restek, Bellefonte, PA, USA) and ENVI-carb X (Supelco).

Standards of ENA, ENA1, ENB, ENB1 and BEA were purchased as powder (premium quality level and/or assay ≥98%) from Sigma-Aldrich (St. Louis, MO, USA). Standard solutions of ENA, ENA1, ENB, ENB1 and BEA were prepared dissolving 10 mg of each compound in 10 mL of MeOH, obtaining stock solutions with a 1-mg·mL^−1^ concentration. Stock solutions were then diluted with pure MeOH in order to obtain the appropriate working solutions. A composite standard working solution was prepared by combining aliquots of each individual working solution and diluting with MeOH to obtain the final concentration of 0.02 mg·L^−1^ for ENA, ENA1, ENB, ENB1 and BEA. All solutions were stored at −20 °C in amber glass vials and darkness before use.

### 3.2. Sampling

The applicability of the method was assessed in ten human urine samples and ten human plasma samples. Samples from volunteer donors were collected in the early morning. Healthy donors were composed of a group of four men and six women, between 25 and 70 years old. All donors signed an informed consent form before the study. The study was conducted in accordance with the World Medical Association’s “Ethical Principles for Medical Research involving human subjects” [30].

Five-milliliter blood and 50-mL urine samples were collected per volunteer. Plasma samples (~2.5 mL) were obtained from whole blood by centrifuging at 1000× *g* for 5 min to pellet blood cells. The supernatant plasma was removed, split into 250-µL aliquots and stored at −80 °C until further use. Urine samples were centrifuged at 2000× *g* for 6 min at room temperature to separate the sediment. Then, sub-samples of 5 mL per volunteer were aliquoted and stored in a dark and dry place at −20 °C until analysis. For analysis, all aliquots were thawed at 4 °C and then allowed to warm at room temperature. The samples with undetectable levels of mycotoxins were used for spiking and recovery studies in the method development. For “undetectable amount”, we did not intend the absence of signal, as the very sensitive detection capability of the instrumentation evidenced some small peaks at retention times very close to those of the analytes, but signals that were not in compliance with the provision established by 2002/657/EC [29] regarding the mean value variation of the area ratio between the two transitions (<MDL). For preliminary experiments and detection/quantification limit evaluation, pools of fresh urine and plasma were used.

### 3.3. Sample Preparation

Sample preparation consisted of an easy sample pretreatment, specific for each body fluid, and a similar cleanup step.

#### 3.3.1. Sample Pretreatments

A 5-mL aliquot of urine was diluted with 500 mL of ultrapure water, and the pH was adjusted to *ca*. 4 (using a pH-meter) with HCl 1 mol·L^−1^ and HCl 0.1 mol·L^−1^. The treated samples were cleaned up using Carbograph-4 cartridges according to the protocol described in the Section 3.3.2.

A 250-µL aliquot of human plasma was treated with 25 mL of MeOH/H_2_O (40/60, *v*/*v*) to achieve plasma deproteinization. Sample was vortexed for 3 min, centrifuged at 3000× *g* for 10 min, and the supernatant was cleaned up using Carbograph-4 cartridges according to the process described in the Section 3.3.2.

#### 3.3.2. Extraction Method

The extraction and cleanup were performed in a single step, and the procedure, valid for both fluids, was performed by SPE using a pre-conditioned Carbograph-4 cartridge. The SPE cartridges were attached onto a vacuum manifold apparatus (Supelco, Bellefonte, PA, USA). First, the cartridges were washed sequentially with 5 mL of CH_2_Cl_2_/MeOH (80/20, *v*/*v*) containing 0.2% of HCOOH, 3 mL of MeOH, 10 mL of 10 mmol·L^−1^ HCl solution and 5 mL of ultrapure H_2_O (at flow rate about 2 mL·min^−1^). Then, the pretreated sample was loaded at a flow rate of 20–25 mL·min^−1^ for urine samples and 2–3 mL·min^−1^ for plasma samples, respectively. The bottle containing the pretreated urine or plasma sample was washed with 100 mL or 10 mL of ultrapure water, respectively, and the washing was passed through the cartridge. Then, 2 mL of MeOH were slowly passed (flow rate of 1 mL·min^−1^) through the cartridge to remove possible interferences without eluting the targeted analytes. Finally, the mycotoxin elution step was performed by passing through the cartridge 10 mL of CH_2_Cl_2_/MeOH (80/20, *v*/*v*) containing 0.2% of HCOOH. The vacuum was adjusted to provide a flow rate of 2–3 mL·min^−1^. The eluate was collected into a 1.4-cm i.d. round-bottom glass vial and evaporated to dryness by a gentle nitrogen stream at 37 °C. The residue was reconstituted either with 500 µL or 250 µL of an ACN/H_2_O (80/20, *v*/*v*) mixture, for urine and plasma samples, respectively; samples were filtered through a 13- mm/0.20-µm nylon membrane syringe filter (Pall Corp., MI, USA) prior to injection into the LC-MS/MS instrumentation.

### 3.4. LC-MS/MS Analysis

For LC-MS/MS analysis, an Ultimate 3000 LC system (Thermo Fisher Scientific, Bremen, Germany) and a TSQ Vantage™ triple-stage quadrupole mass spectrometer (Thermo Fisher Scientific) connected via an electrospray (ESI) source operating in positive ionization mode were used for the determination of the analytes. The LC-MS/MS system was managed by the Xcalibur software (v.2.1, Thermo Fisher Scientific, Bremen, Germany).

The LC system consisted of a binary pump connected to a degasser, a thermostated microwell plate autosampler set at 14 °C and a thermostated column oven maintained at 30 °C. The injection volume was 5 µL. The separation was achieved by a Hypersil Gold analytical column (150 mm × 2.1 mm i.d., 3 µm particle size) preceded by a SecurityGuard Hypersil Gold pre-column (4 mm × 2.1 mm i.d., 5 µm particle size), both supplied by Thermo Fisher Scientific. The mobile phase consisted of H_2_O (A) and MeOH (B), both containing 5 mmol·L^−1^ ammonium formate and 0.1% (*v*/*v*) HCOOH. Gradient elution was started isocratically with 50% B for 1 min. Then, B was linearly increased to 99.5% within 6.5 min and kept constant for 3 min. Finally, B was decreased linearly to 50% in 0.5 min and equilibrated for 5 min. The flow rate was set at 300 µL·min^−1^.

Mass calibrations and resolution adjustments on the resolving lens and quadrupoles were automatically performed using the manufacturer solution introduced by infusion pump at a 5-µL·min^−1^ flow-rate. In order to optimize the acquisition parameters for each mycotoxin, 1 ng·µL^−1^ of individual standard solutions prepared in starting mobile phase was infused into the instrument. The [M+NH_4_]^+^ ions were selected by the first quadrupole and fragmented in the collision cell with the appropriate CE. According to the European Union criteria established for contaminants in food [29], from the MS/MS full-scan spectra, two suitable transitions were selected for acquisition in SRM mode. The selected precursor ion, the two most intense product ions and the optimized SRM parameters (CE and S-lens) of each analyte are presented in Table 1.

Regarding the optimization of general mass spectrometric parameters, the source settings were as follows: 3.2 kV for spray voltages, 280 °C for vaporizer temperature, 220 °C for capillary temperature, 50, 1 and 25 (arbitrary units) for sheath gas pressure, ion sweep gas pressure and auxiliary gas pressure, respectively.

### 3.5. Method Performance

Performance characteristics of the method included the evaluation of linearity, recovery (yield), process efficiency (PE), trueness, precision (intra- and inter-day precision), MDLs and MQLs.

#### 3.5.1. Linearity

Linearity was evaluated by preparing three sets of calibration curves (standard calibration curve, urine and plasma matrix-matched calibration curves) at six concentration levels. Solutions for the standard calibration curve were prepared by diluting composite standard working solution into the solvent over the range 0.04–4.0 ng·mL^−1^ (0.2–20 pg injected). Plasma and urine matrix-matched calibration curves were prepared by spiking the individual blank samples with the composite standard working solution in the range of 40–4000 ng·L^−1^. These samples were treated according to the extraction procedure described in Section 3.3. Each calibration curve was constructed in triplicate during three consecutive days, and results were averaged.

For each analyte, the combined ion current profile for the selected transitions was extracted from the LC-SRM dataset; the resulting traces were smoothed by applying the automatic processing smoothing method (Xcalibur) using Gaussian type (7 points). Calibration curves were constructed by plotting the peak area sum of the two transitions *versus* the mycotoxin concentration. Unweighted regression lines for standard and matrix-matched calibration curves were calculated. The latter calibration curves were used for quantification.

#### 3.5.2. Recovery and Process Efficiency

Recovery was assessed by comparing the area of peaks obtained for the analytes added to the sample before and after the extraction procedure. The combined effects of signal suppression/enhancement, *i.e.*, ME, recovery and PE, were determined by comparing the slopes of the standard calibration line (a_standard_) with that of the matrix-matched calibration line (a_matrix_) [28]. The calculation of the PE was performed according to the formula: PE (%) = (a_matrix_/a_standard_) × 100.

#### 3.5.3. Detection and Quantification Limits

Instrumental limits of detection (ILODs) and instrumental limits of quantification (ILOQs), as well as MDLs and MQLs for each matrix were calculated to evaluate the optimized LC-MS/MS method. For the calculation of detection limits, the standard deviation of the response (σ) was divided by the slope of the calibration curve (*S*), via the formula: detection limit = 3 σ/*S*. In the same way, quantification limits were estimated according to the formula: quantification limit = 10 σ/*S*. ILOQs, ILODs, MDLs and MQLs were calculated employing data generated from regression statistic performed using standard calibration and matrix-matched calibration, respectively. These values were verified by adding to the samples the concentration obtained by the reported procedure and adjusted by direct sample injection. The sum of the ion currents of the SRM transitions was considered to determine ILOQs, while the less intense transition was considered to evaluate ILODs. Both transitions were considered for the estimation of MQLs and MDLs (see Section 2.3.2).

#### 3.5.4. Trueness and Precision

Trueness and precision were evaluated for each matrix at three concentration levels: MQL, 2.5 times MQL and 10 times MQL. To achieve this goal, blank samples of urine and plasma were fortified with an appropriate volume of the composite working standard solution to obtain the above-mentioned concentration levels. Trueness was assessed by measuring the peak area of the spiked samples and comparing this result with that obtained from the matrix-matched calibration curve; the result was expressed as the percentage. The precision of the method was determined as within laboratory precision using the average trueness. Six replicates for each concentration level were analyzed in one day to evaluate intra-day precision. Another three replicates for each concentration level were prepared and analyzed on five additional days to estimate the inter-day precision. The method precision was expressed as the RSD of replicate measurements.

Statistical comparisons were performed by ANOVA (*p* = 0.05).

## 4. Conclusions

In this study, rapid and sensitive methods for the determination of ENs and BEA in human biological fluids were developed and fully validated. The two methods differ only for the pretreatment step, which is sample-type specific. Regarding the plasma matrix, the MDLs of the present method were 5–10-times lower than those reported in a previous work for the determination of ENs and BEA in pig plasma [17].

The analysis of 10 subjects, who followed their normal diet, basically a Mediterranean diet, rich in cereals, with no particular indications, did not show any parent mycotoxin either in plasma or urine. This fact does not mean that ENs and BEA cannot pose a problem for human exposure, but only that this problem is of minor impact in countries where mycotoxin level controls are extensive.

In any case, the proposed extraction coupled to the LC-MS/MS method offered a reliable quantitative analysis of target mycotoxins. This method may be used also for *in vivo* studies where samples could be collected, stored and processed even immediately, as the activated GCB cartridges are stable for weeks. Blood samples are less easily processed than urine samples, as plasma must be separated from cells and deproteinized. However, the subsequent extraction procedure does not change with respect to stored samples.

This is the first report on the presence (absence) of emerging *Fusarium* mycotoxins in urine and plasma of humans. Owing to the fact that target mycotoxins were not detected in any sample, it would be of great interest to characterize the routes of metabolization of each emerging *Fusarium* mycotoxin in humans for a better screening of human exposure in future biomonitoring studies, which would therefore also include metabolite determination.
